# Accurately Assessing Teacher ADHD-Specific Attitudes Using the Scale for ADHD-Specific Attitudes

**DOI:** 10.1177/10870547231153938

**Published:** 2023-02-26

**Authors:** Sarah Mulholland, Therese M. Cumming, Jihyun Lee

**Affiliations:** 1 UNSW, Sydney, Australia

**Keywords:** ADHD, teacher, attitudes, Attention Deficit Hyperactivity Disorder, predictors

## Abstract

**Objective:**

The aim of this study is therefore twofold, first to accurately examine the ADHD-specific attitudes of New South Wales Government school in-service teachers using the Scale for ADHD-Specific Attitudes (Authors 2016), and second, to determine if any of their socio-demographic features could predict their attitudes

**Method:**

Exploratory factor analysis found a 5-factor structure, and multiple regression analysis was performed to establish the existence of groups of variables with respect to teacher attitudes towards ADHD-type behaviours and any demographic predictors of teacher attitudes.

**Results:**

The final regression model found significant predictors of each factor with R2 values ranging from .007 to .147.

**Conclusion:**

This study illustrated that teachers had generally positive attitudes towards students who display ADHD-type behaviours, however, they found the externalised behaviours of ADHD irritating in the classroom and found teaching students with ADHD-type behaviours difficult, and teachers want more information about ADHD and how to manage it in the classroom.

## Introduction

Globally, approximately 7.2% of school-aged children (American Psychiatric Association, 2022) are diagnosed with attention-deficit/hyperactivity disorder (ADHD), a neurodevelopmental disorder with three presentations (inattention, impulsive/hyperactive, or combined). While differences in attention, hyperactivity, and/or impulsivity are typical, the main symptoms of ADHD must be found at a persistent and significant level which interferes with a person’s functioning or development for a diagnosis to be made (American Psychiatric Association, 2022). ADHD-type behaviors become more apparent when a child begins formal education and they can have significant educational implications for students, including lower academic achievement (Barkley, 2015) and motivation to avoid failure rather than to achieve success (Olivier & Steenkamp, 2004), and can often mask comorbid learning disabilities (Barkley, 2015) and mental health disorders (American Psychiatric Association, 2022). Specifically, students with ADHD are three times more likely to have a specific learning disability in reading, writing, or mathematics than their peers without ADHD (Carroll et al., 2005) and so masking of comorbid disorders compound the impact of ADHD on student educational outcomes. There are approximately two students with a diagnosis of ADHD in every classroom (of about 30 students) and teachers are often the first to refer students for assessments (Mulholland et al., 2015). Thus, it is essential to understand the ADHD-specific attitudes of teachers, as attitude directly impacts behavioral intention, which in turn affects teacher behavior in the classroom (van Aalderen-Smeets et al., 2012). In understanding teacher attitudes, professional learning may be targeted to both improve teacher ADHD-specific attitudes and teacher self-efficacy so that teachers can initiate and maintain the provision of appropriate adjustments, and support these students to have positive school experiences and educational outcomes.

## Literature Review

Researchers have investigated teacher ADHD-specific attitudes for nearly 30 years. Of the existing studies many purport to explore attitudes, yet simply examine teacher knowledge (e.g., Bekle, 2004; Jerome et al., 1994, 1999), investigate teacher stress and ADHD stigma (Bell et al., 2011; Greene et al., 2002, respectively), use a traditional model of attitude to develop their research instrument (Kos, 2008), or attempt to understand teacher attitudes based on a global attitude score (Anderson et al., 2012). Jerome et al. (1994), aimed to examine teacher ADHD-specific attitudes through a self-designed research instrument consisting of 20 true or false questions which assessed the knowledge teachers had with respect to ADHD. Although the title of this study suggested it would investigate teacher attitudes toward ADHD, it did little more than examine the factual knowledge and misinformation teachers held. Various other studies share similar deficits in their research instrument choice or design, using factual statements, having limited ability to assess teacher attitudes, and instead predominantly assessing teacher ADHD-specific knowledge and/or misperceptions (e.g., Alanazi & Al Turki, 2021; Bekle, 2004; Rodrigo et al., 2011; Sciutto et al., 2000; Soriano Ferrer & Echegaray-Bengoa, 2021).

Further to these studies, various researchers have used traditional models of attitude including the Theory of Planned Behavior (TPB) (Ajzen, 1985, 2001) to design their research instruments (e.g., Alabd et al., 2018; Anderson et al., 2012; Dort et al., 2020; Kos, 2008; Youssef et al., 2015). These models of attitude describe the well-established link between attitude and behavior, that attitude informs the intention to complete the target behavior and is followed by the occurrence (or not) of that behavior. Thus, these models use a target behavior (such as teaching children with ADHD) as the attitudinal object (the entity toward which the attitude is directed). Due to the reliance on the traditional models of attitude, these studies have the same deficit in attempting to assess an accurate picture of teacher attitudes of ADHD, students who exhibit ADHD-type behaviors, and how to educate them, as they primarily focus on teacher attitude toward performing a specific behavior such as teaching students with ADHD.

While many studies either purport to examine the ADHD-specific attitudes of teachers, or use traditional models of attitude to do so, there are studies that have investigated a more multi-faceted view of attitude with respect to ADHD. Mulholland et al. (2015) designed a research instrument based on the more holistic theoretical framework of attitude proposed by van Aalderen-Smeets et al. (2012) to examine the ADHD-specific knowledge and attitudes held by NSW metropolitan government school teachers. To assess attitudes, 30 questions with a 6-point Likert scale were used. The survey found a four-factor structure that included the factors (a) *feelings about teaching students who exhibit ADHD-type behaviors*, (b) *knowledge and training regarding ADHD*, (c) *desire for better training regarding ADHD*, and (d) *beliefs about ADHD and its associated behaviors.* The study by Mulholland et al. is limited due to the small sample size (111 participants) and therefore its findings were unable to be generalized to a broader community. However, the instrument was improved upon by Mulholland (2016) and was found to be valid and reliable.

This improved research instrument has been used in subsequent studies. Cueli et al. (2022) examined the knowledge and attitudes of 417 pre-service and 170 in-service teachers from Spain. They highlighted that more than two-thirds of their pre-service (73%) and in-service (86.5%) teachers found ADHD-type behaviors irritating in the classroom. However, both groups tended to agree with the positive feelings and beliefs statements and disagree with the negative feelings and belief statements. Concerningly, the study also found that just over half (55%) of in-service teachers and 36% of pre-service teachers felt that they could effectively teach students with ADHD. However, 100% of pre-service and 98% of in-service teachers in this study reported that they would like to know more about ADHD.

Similarly, Dessie et al. (2021) used the attitudes section, the Scale for ADHD Specific Attitudes, of the aforementioned research instrument (Mulholland, 2016), to assess the ADHD-specific attitudes of 636 in-service teachers in Gondar, Ethiopia. They found that 84.1% of teachers held favorable attitudes toward ADHD and that the likelihood of reporting favorable attitudes were 1.85 times higher when the teachers had previous experience teaching a child with ADHD. Despite this study using an instrument that investigates a more holistic view of teacher ADHD-specific attitude (rather than simply investigating teacher behavior toward teaching students with ADHD), the study provided a very rudimentary analysis, which included a global attitudes score and only two socio-demographic data points (experiencing teaching a student with ADHD, and the source of teacher ADHD-specific knowledge). As such, it provides limited insight into the ADHD-specific attitudes of its participants.

While there has been an increase in literature investigating teacher attitudes toward ADHD recently, few investigate the influence of socio-demographics on the ADHD-specific attitudes of participants. Of the studies that investigated socio-demographic data as predictors of ADHD-specific attitudes, most examined the difference between pre-service and in-service teachers (e.g., Bekle, 2004; Cueli et al., 2022; Jerome et al., 1999) and found little difference between the two groups. Additionally, Mulholland et al. (2015) found that experience (measured in years teaching) was not a predictor of most ADHD-specific attitudes. However, it was found to be a significant predictor of the factor *Feelings About Teaching Students Who Exhibit ADHD-Type Behaviors*, which included items indicating negative feelings toward students with ADHD (e.g., students with ADHD cause teachers to experience stress, and interfere with their ability to effectively teach their class). This suggested that as teacher’s experience increased, they were more likely to agree with items about this factor, a result that was substantiated by Dort et al. (2020).

Only a handful of studies investigated the influence of other socio-demographic features on ADHD-specific attitudes. Results suggested that teaching students with ADHD diagnoses increased positive attitudes toward them (Amha & Azale, 2022; Dessie et al., 2021), as did watching mass media as an information source about ADHD (Dessie et al.). Furthermore, Cueli et al. (2022) found that as teachers aged, their ADHD-specific attitudes became more negative, while having a close relationship with a student who had ADHD, having a relative with ADHD, or having ADHD themselves led to more positive opinions of their students with ADHD (Flavian & Uziely, 2022). Finally, special education teachers were more likely to have more positive global attitudes scores (Flavian & Uziely), which contradicted an earlier study by Mahar and Chalmers (2007) who found that mainstream teachers were significantly more likely to strongly agree with the statement, “Students diagnosed with ADHD can learn successfully and effectively in the regular classroom” than their special education counterparts. Unsurprisingly, special education and learning and support teachers were found to be more likely to agree (to some degree) that they feel knowledgeable about ADHD-type behaviors, feel they have received adequate training with respect to the classroom management of ADHD-type behaviors, and feel effective in doing so than their mainstream counterparts (Mulholland et al., 2015).

A thorough review of the literature revealed limited studies that accurately assessed teacher ADHD-specific attitudes and fewer still which examined the socio-demographic predictors of these attitudes. The present study examined the attitudes held by NSW government school teachers with respect to ADHD, students who exhibit ADHD-type behaviors and the teaching thereof, and socio-demographic features that could predict these attitudes.

## Theoretical Framework

As mentioned above, theoretical models of attitudes that have been developed in psychology in general, describe the well-established link between attitude and behavior, that attitude informs intention and behavior (Ajzen, 1985, 2001). As such, the attitudinal object (or focus of attitude) used in these traditional models is a desired or expected behavior (in many studies of ADHD, it is appropriate classroom management of or educational instruction for students with ADHD). Thus, these traditional models are inappropriate frameworks to use and may not be optimal when investigating teacher attitudes toward ADHD, students who display ADHD-type behavior and the teaching thereof. In order to complete such investigations, van Aalderen-Smeets et al. (2012) developed a new theoretical framework. Their framework acknowledges that attitude is a multi-dimensional concept and research should investigate various dimensions to form a clear picture of attitudes. The new framework does not require a behavior as the attitudinal object and suggests that both behavior and behavioral intention should be seen as theoretically different from attitude. Van Aalderen-Smeets et al. (2012) identified three main dimensions of attitude: cognitive beliefs, affective states, and perceived control, each of which consist of subcategories that refer to both personal and professional attitudes. This theoretical framework was the foundation for the research instrument on which this study is based.

## Importance of the Present Study

The objective of this paper was twofold. The first goal of this paper was to accurately assess the ADHD-specific attitudes (defined as a feeling or position with regard to ADHD, students who display ADHD-type behaviors, and the teaching thereof) held by NSW Government School in-service teachers using the Scale of ADHD-Specific Attitudes (Mulholland, 2016). While there has been an increase in academic research around this topic, there are few studies that examine the ADHD-specific attitudes of teachers in Australia. Around the world and in Australia, many studies purport to assess attitudes using questionnaires featuring factual questions and, as such, merely examine teachers’ knowledge and misperceptions of ADHD. Additionally, studies that do examine ADHD-specific attitudes, use traditional models of attitude and merely examine attitude toward a particular teacher behavior such as using effective teaching strategies to support students with ADHD. Unfortunately, neither of these approaches can provide an accurate picture of ADHD-specific attitudes.

Given the strong link between attitudes, behavioral intention, and behavior and the limitations in many of the previous studies, there is a strong need for a larger scale investigation into ADHD-specific attitudes held by NSW Government school in-service teachers.

The second goal of this paper was to determine if any of the participants’ socio-demographic data could be used to predict their ADHD-specific attitudes, as there is limited literature that does this, and studies which do, generally compare the ADHD-specific attitudes of pre-service and in-service teachers. This study was somewhat unique in its approach, as it investigated the predictive power of various socio-demographic aspects of the participants on their ADHD-specific attitudes. With a greater understanding of teacher ADHD-specific attitudes, policy makers and school leadership may further target professional development to improve teacher attitudes, and thus have a positive impact on teacher behavioral intention and teacher behavior with respect to students who display ADHD-type behaviors. The research questions the present study aimed to answer were:

1. What are the attitudes of NSW Government school in-service teachers toward ADHD, the students who exhibit ADHD-type behaviors, and the teaching thereof?
2. Are there any socio-demographic predictors of teachers’ ADHD-specific attitudes?

## Method

### Ethical Approval

Ethical approval for this study was obtained from the authors’ affiliated institution and the NSW Department of Education. In addition to ethical approval, prospective participants were provided with a participant information form consistent with the National Statement on Ethical Conduct in Research Guidelines, Section 2.2.5 (Australian Government National Health and Medical Research Council, 2018).

### Participants

The sample of this study was comprised of teachers from government schools in New South Wales (*N* = 596). To recruit participants, principals from each of the 2,245 government schools in NSW were sent a recruitment email explaining the study and asking for the school’s participation. One hundred fifty-four (154) principals agreed to have their school involved in the study. Subsequently, they were sent a second email containing information for participants, and a link to the online survey, or information regarding the hard copies of the survey. The survey was distributed to staff by their principal. The number of NSW government school teachers who received the request to participate in the study totaled 1,800. Teachers completed the survey voluntarily. The sample of teachers were recruited from Greater Sydney Region, including metropolitan Sydney (50.67%), regional cities (20.64%), and rural areas (24.83%). They were teachers in primary schools (48.15%), high schools (30.70%), and a variety of other educational settings (21.15%). There were more female teachers (*n* = 457) than male teachers (*n* = 132). The average age of the sample was 44 years old. The average number of years in teaching was 17. Nearly all teachers in the sample (97%; 578 out of 596) reported having taught a student with ADHD behaviors. Most of them (83%; 493 out of 596) also reported that those students had been diagnosed with ADHD. Further, the majority of the teachers in the sample (77%; 456 out of 596) reported that they were currently teaching students with ADHD-type behaviors. Finally, the participants reported that the average number of students who exhibited ADHD-type behaviors in their classroom was 4.67. Table 1 lists more detailed information about the study participants.

**Table 1.** Participant Information About Demographics and Teaching of ADHD Students (*N* = 596).

Teacher characteristics	Distributions
Gender	
Male	132
Female	457
Missing value	3
Age	
Range	21–69
Mean	44.07
Mode	50
Missing value	13
Birthplace	
Australia	491
Other	96
Missing value	5
Teaching experience (number of years)	
Range	1–45
Mean	17.39
Mode	30
Missing value	6
Subject taught	
Special education/learning support	67
Other	518
Missing value	7
Degree	
Bachelor’s degree	303
Diploma	55
Graduate diploma	101
Master’s degree	121
Ph.D.	3
Missing data	9
School’s geological area	
Sydney metropolitan area	273
Greater Sydney area (excluding Sydney metropolitan area)	29
Small regional city	52
Large regional city	71
Rural area	148
Missing	19
School type	
Infants	13
Primary (K-6)	234
Primary with opportunity classes	7
Primary with special needs classes	46
Secondary—Comprehensive	154
Secondary—Selective	3
Secondary school—Collegiate (7–10)	7
Secondary school—Collegiate (11–12)	19
School for specific purposes	49
Central and community school (K-12)	12
Central and community special needs school (K-12)	6
Distance education	5
Intensive English Center	4
Primary school with preschool	5
Environmental Education Center	5
Other	1
Missing	22
School gender	
Boys’ school	11
Girls’ school	21
Co-educational school	536
Missing	24
Have you taught any student with ADHD behaviors?	
Yes	578
No	6
Missing	8
Did those students have an ADHD diagnosis?	
Yes	493
No	74
Missing	25
Do you currently teach students with ADHD behaviors?	
Yes	456
No	114
Missing	22
How many students with ADHD behaviors do you currently teach?	
Range	0–51
Mean	4.67
Mode	2
Missing value	149
How many students have a current diagnosis?	
Range	0–25
Mean	2.22
Mode	0
Missing value	177

### Research Instrument

The ADHD-Specific Knowledge and Attitudes of Teachers questionnaire (ASKAT) (Mulholland, 2016) was used in this study. The questionnaire consisted of four sections, however, the participants’ responses in Sections A and C were analyzed and presented for the present study. Section A was comprised of sociodemographic questions, that is, age, gender, experience, teaching subject, birthplace, highest education degree, experience teaching students with ADHD, school location, and school type. Additionally, the knowledge scores for each participant from Section B of the ASKAT were used a point of demographic data for analytical purposes, that is, their knowledge score was added to the analysis to determine if it was predictive of teacher ADHD-specific attitudes. Section B contained 20 items that tested teachers’ knowledge about ADHD, using the true/False/I don’t know answer system to avoid inflated scores due to guessing. The scores ranged from a minimum of 0% to a maximum of 100%, with the mean score of 73.32% and median score of 75% (Mulholland et al., 2022).

Section C contained the items of the Scale for ADHD-Specific Attitudes (SASA), consisting of 30 questions related to teacher attitudes toward (a) ADHD itself, (b) students who display ADHD-type behaviors, and (c) their views on the teaching of these students. The response category was a 6-point Likert-scale. The ASKAT has been shown to be valid, reliable, and generalizable (more specifically generalizable to anglophone countries) (Mulholland, 2016). Mulholland used exploratory factor analysis to determine both the reliability through an appropriate level of explained variance (proposed by Field, 2009) and validity through Cronbach alpha values to examine the instrument’s internal consistency.

## Analysis

Descriptive statistics such as frequency analysis (FA) and means analysis were carried out on the item responses to the SASA to examine the respondents’ agreement/disagreement on their ADHD-specific attitudes. Then, exploratory factor analysis (EFA) was performed to extract factors of teacher attitudes toward ADHD and to examine the underlying dimensionality of the items by grouping them into meaningful subsets of items.

Finally, multiple regression analysis was performed to identify teachers’ socio-demographic characteristics that predict their ADHD-specific attitudes. The independent variables were entered into the regression models in the data format found in Table 2.

**Table 2.** Data Format for Multiple Regression.

Variable	Type	Description
Age	Continuous	Participant age in years
Student’s diagnostic status	Dichotomous	Indicating whether the participant was currently teaching a student who has an ADHD diagnosis (1: yes, 0: no)
School type	Dichotomous	Indicating whether the participant is teaching in a school for specific purposes (SSP; 1: SSP, 0: other)
Knowledge score	Continuous	Variable drawn from the participants’ score on the items related to ADHD-specific knowledge
Current number	Continuous	Representing the number of students with ADHD-type behaviors a participant was currently teaching
Subject taught	Dichotomous	Indicating if whether the participant was a Special Education or Learning Support Techer (1: special education/learning support, 0: other)
Degree	Dichotomous	Indicating a participant’s educational highest degree that s/he have obtained (1: more than Bachelor’s degree, 0: Bachelor’s degree)

## Results

### Frequency Analysis

Data from the SASA was analyzed to examine the attitudes NSW teachers held toward ADHD itself and students who exhibit ADHD-type behavior (see Table 3). More than 90% of participants agreed (an aggregate of the responses from strongly agree, agree, and somewhat agree) that ADHD is a valid diagnosis while more than half of the participants indicated that they felt ADHD is over-diagnosed. In general, teachers held favorable attitudes toward teaching students with ADHD. This is indicated by 71.08% of participants agreeing at least to some degree with the statement “students who exhibit behaviors associated with ADHD are rewarding to work with,” while more than three quarters (77.13%) of the respondents agreed to some degree that “students who exhibit behaviors associated with ADHD bring new perspectives to the topics” they are teaching. Similarly, almost three quarters (72.77%) of the respondents disagreed (an aggregate of the responses from strongly disagree, disagree, and somewhat disagree) with the item “I dislike teaching classes that contain students who display ADHD-type behaviors.” However, the majority (65.97%) believed that these “students interfered with their ability to effectively teach their class.” In addition to this, about 40% of participants did not feel it was “easy to implement educational accommodations for student with ADHD.”

**Table 3.** Agreement/Disagreement Rating on the Scale for ADHD-Specific Attitudes (SASA).

Question	Strongly disagree	Disagree	Somewhat disagree	Somewhat agree	Agree	Strongly agree	Unanswered
Students who exhibit behaviors associated with ADHD are rewarding to work with							
	2.65	8.32	16.64	31.76	30.81	8.51	1.32
Students who exhibit behaviors associated with ADHD interfere with my ability to effectively teach my class							
	3.97	10.59	17.39	42.34	18.90	4.73	2.08
Students who exhibit behaviors associated with ADHD perform well in some subjects and not others							
	0.76	2.46	7.18	31.95	46.12	8.88	2.65
Students who exhibit behaviors associated with ADHD have no excuse for their poor behavior if they do not have a formal diagnosis							
	35.73	43.10	13.42	4.35	1.51	0.57	1.32
Students who exhibit behaviors associated with ADHD misbehave because they don’t want to follow the set rules							
	27.03	38.56	15.88	13.23	2.65	0.95	1.70
Students who exhibit behaviors associated with ADHD need more structure and discipline, not assistance with their academic work							
	14.18	26.84	20.79	20.04	11.91	3.78	2.46
Students who exhibit behaviors associated with ADHD bring new perspectives to the topics I am teaching							
	2.08	6.24	12.67	42.91	27.79	6.43	1.89
Students who exhibit behaviors associated with ADHD need to try harder to focus on their school work							
	6.43	20.79	21.93	34.40	11.15	3.02	2.27
I believe ADHD is over diagnosed							
	5.10	16.07	26.47	36.67	9.45	4.73	1.51
I believe ADHD is a valid diagnosis							
	0.76	0.95	3.97	21.36	51.80	19.66	1.51
I believe ADHD is an excuse for poor parenting							
	32.14	31.19	19.28	11.53	3.02	0.95	1.89
I believe children who exhibit ADHD-type behaviors are deliberately misbehaving							
	31.57	41.21	16.07	6.81	2.08	0.38	1.89
I would refer a student who exhibited ADHD-type behaviors in my classroom to the school counselor for a possible ADHD assessment							
	1.32	4.16	3.97	13.61	49.15	27.03	0.76
I would like to know more about ADHD and its associated behaviors							
	1.51	2.46	3.59	18.71	48.77	24.20	0.76
I would like to have more information about classroom interventions to assist me with educating students who display ADHD-type behaviors							
	1.13	1.70	1.70	14.93	43.67	35.92	0.95
I find behaviors associated with ADHD irritating in the classroom							
	2.84	12.85	12.67	46.69	18.71	5.48	0.76
I find students who exhibit ADHD-type behaviors rude							
	8.70	24.39	24.95	31.76	6.81	1.70	1.70
I find it challenging to teach students who exhibit behaviors associated with ADHD							
	0.57	4.16	7.56	41.59	34.22	10.59	1.32
I find it rewarding to see the accomplishments of students who display ADHD-type behaviors							
	0.57	0.57	0.57	17.39	47.83	31.57	1.51
I find students who display ADHD-type behaviors cause me to experience stress							
	2.08	13.23	16.45	44.05	18.15	5.10	0.95
When it comes to differentiation, I feel ADHD is a benefit to the growth of my teaching skills							
	1.32	1.32	9.64	38.75	35.73	11.53	1.70
When it comes to differentiation, I feel I don’t have the time							
	21.93	30.43	21.36	17.39	4.16	3.02	1.70
When it comes to differentiation, I feel I already change my lessons and teaching styles							
	1.51	2.08	4.91	31.00	41.78	17.39	1.32
When it comes to differentiation, I feel educational accommodations for students with ADHD-type behavior are easy to implement in a general education classroom							
	3.02	14.18	23.06	34.22	21.36	2.84	1.32
I feel I am knowledgeable about ADHD-type behaviors							
	1.13	7.75	14.18	41.40	24.39	5.10	6.05
I feel I am knowledgeable about classroom interventions to manage misbehavior							
	0.95	4.91	10.59	41.78	31.19	8.13	2.46
I feel I have received adequate professional development about managing ADHD-type behaviors							
	7.75	20.79	25.71	28.54	13.42	2.84	0.95
I feel I can effectively teach students who exhibit behaviors associated with ADHD							
	1.32	4.91	14.56	44.61	27.98	5.48	1.13
I feel I want to be more effective teaching students who display ADHD-type behaviors							
	0.38	1.51	2.46	24.39	44.61	25.52	1.13
I feel I dislike teaching classes that contain students who display ADHD-type behaviors							
	18.71	30.43	23.63	20.23	3.78	1.32	1.89

Although teachers generally have favorable attitudes toward the students who exhibit ADHD-type behaviors, they generally have negative attitudes toward ADHD-type behaviors themselves. The majority of teachers (70.88%) agreed at least to some degree that they find “behaviors associated with ADHD irritating in the classroom,” and just over two-thirds (67.30%) of the respondents agreed to some degree that these students cause them to “experience stress.” An overwhelming majority (94.52%, an aggregate of the responses, strongly agree, agree, and somewhat agree) of teachers felt that they would “like to be more effective when teaching students who exhibit ADHD-type behaviors,” while only 44.80% of teachers agreed at least to some degree that they “had received adequate professional development about managing ADHD-type behaviors.”

### Exploratory Factor Analysis (EFA)

EFA was conducted on the item responses of the Scale for ADHD-Specific Attitudes (SASA, 30 items). A five-factor structure was identified with the eigenvalues of 5.425 (*Negative consequences of ADHD-type behaviors*), 2.330 (*Desire for professional learning*), 1.911 (*Feeling of knowledgeability*), 1.586 (*Negative beliefs about ADHD*), 1.105 (*Positive consequences of ADHD-type behaviors*). These five factors accounted collectively for 65.03% of the total variance of the item responses, above the 60% threshold noted by Hair et al. (2010) for a satisfactory factor solution. Bartlett’s test of sphericity (3,522.023, *p* < .001) and Kaiser-Meyer-Olkin’s (KMO) measure of sampling adequacy (0.832) indicated that factor analysis was appropriate. The factor loadings are for most part greater than 0.50 with only one exception of 0.355. According to Hair et al. (2010), items that demonstrated standardized factor loadings greater than 0.30 are considered having contributed to the corresponding factor. The Cronbach’s alpha values for the five factors are greater than .75 and are deemed reasonably high. Table 4 presents the items, standardized factor loadings, and Cronbach’s alpha values of each factor.

**Table 4.** Exploratory Factor Analysis.

Factor and items	Factor loading
Factor 1: Negative consequences of ADHD-type behaviors (Cronbach’s α = .811)	
I find behaviors associated with ADHD irritating in the classroom.	0.734
I find students who display ADHD-type behaviors cause me to experience stress.	0.727
Students who exhibit behaviors associated with ADHD interfere with my ability to effectively teach my class.	0.581
I find students who exhibit ADHD-type behaviors rude.	0.525
I feel I dislike teaching classes that contain students who display ADHD-type behaviors.	0.522
When it comes to differentiation, I feel I don’t have the time.	0.355
Factor 2: Desire for Professional Learning (Cronbach’s α = .893)	
I would like to know more about ADHD and its associated behaviors.	0.945
I would like to have more information about classroom interventions to assist me with educating students who display ADHD-type behaviors.	0.887
Factor 3: Feeling of knowledgeability (Cronbach’s alpha = .827)	
I feel I am knowledgeable about classroom interventions to manage misbehavior.	0.844
I feel I am knowledgeable about ADHD-type behaviors.	0.794
I feel I can effectively teach students who exhibit behaviors associated with ADHD.	0.735
Factor 4: Negative beliefs about ADHD (Cronbach’s α = .756)	
I believe children who exhibit ADHD-type behaviors are deliberately misbehaving.	0.791
Students who exhibit behaviors associated with ADHD misbehave because they don’t want to follow the set rules.	0.674
I believe ADHD is an excuse for poor parenting.	0.630
Students who exhibit behaviors associated with ADHD have no excuse for their poor behavior if they do not have a formal diagnosis.	0.553
Factor 5: Positive consequences of ADHD-type behaviors (Cronbach’s α = .774)	
When it comes to differentiation, I feel ADHD is a benefit to the growth of my teaching skills.	0.710
Students who exhibit behaviors associated with ADHD bring new perspectives to the topics I am teaching.	0.674
I find it rewarding to see the accomplishments of students who display ADHD-type behaviors.	0.638
Students who exhibit behaviors associated with ADHD are rewarding to work with.	0.584

### Multiple Regression Analysis

The five factors extracted via EFA were shown to be a good representation of the item responses of the study’s participants. Next, multiple regression analyses was performed with each of those five factors as the dependent variables, to examine if teachers’ socio-demographic variables and their ADHD-specific knowledge could predict their attitudes toward ADHD. The assumptions for multiple regression analysis were first examined. The Durbin-Watson statistic results, ranging from 1.8 to 2.2 for each of the five regression models indicated that the results could be used for making inferences to the wider population. The variance inflation factor (VIF) values, ranging from 0.95 to about 1.0, showed no multicollinearity across the independent variables. A final set of regression models, which contain statistically significant predictors only, are presented in Table 5. That is, the regression models were run as the final set of models with statistically significant predictors only after the initial regression models with the predictor variables (mentioned in the Methods section) were run.

**Table 5.** Final Multiple Regression Model on Each of the Five Factors.

Model	*B*	s.e. in *B*	*β*	Significance (*p*)	*R*^2^ value
Factor 1					.045
Constant	−.370	0.189		.050*	
Age	0.015	0.004	.193	.000**	
Student’s diagnostic status	−.361	0.127	−.134	.005**	
Factor 2					.007
Constant	0.117	0.038		.002**	
School type (SSP vs. O)	−.252	0.138	−.086	.068	
Factor 3					.147
Constant	−1.835	0.222		.000**	
Student’s diagnostic status	0.490	0.115	.189	.000**	
Knowledge score	0.020	0.003	.312	.000**	
Factor 4					.067
Constant	0.821	0.242		.001**	
Current number with ADHD behaviors	0.029	0.010	.149	.004**	
Knowledge score	−.014	0.003	−.220	.000**	
Factor 5					.101
Constant	0.076	0.309		.806	
Age	−.017	0.006	−.239	.009**	
Subject taught	0.316	0.123	.122	.017*	
Degree	0.154	0.077	.094	.047*	
Knowledge score	0.006	0.003	.103	.035	

** *p* ≤ .01. *p = .05.

#### Multiple Regression of Factor 1: Negative Consequences of ADHD-Type Behaviors

The final regression model indicated that age and student diagnostic status were significant predictors of teachers’ responses to the items that formed the factor labeled as *negative consequences of ADHD-type behaviors* (*R*^2^ = .045). As can be seen, the older respondents (*β* = .193; *p* < .001) were predicted to have stronger views on *negative consequences of ADHD-type behaviors* (while controlling for the other variable in the model). The respondents who had taught a student with an ADHD diagnosis (*β* = −.134; *p* < .005) were less likely to endorse *negative consequences of ADHD-type behaviors* (while controlling for the other variable in the model).

#### Multiple Regression of Factor 2: Desire for Professional Learning

The regression model for the second factor, *desire for professional learning* was not explained by our chosen independent variables (*R*^2^ = .007).

#### Multiple Regression of Factor 3: Feeling Knowledgeable About ADHD

The regression model illustrated that both participants’ knowledge about ADHD score and whether they had taught a student with an ADHD diagnosis were significant predictors of their responses to the items which formed the factor, *feeling knowledgeable about ADHD* (*R*^2^ = .147). The participants’ *feeling knowledgeable about ADHD* were predicted to be stronger if they had a higher score on the knowledge test (*β* = .312; *p* < .001) and if they had taught a student with an ADHD diagnosis (*β* = .189; *p* < .001).

#### Multiple Regression of Factor 4: Negative Beliefs About ADHD

Concerning the factor, *negative beliefs about ADHD*, the regression model (*R*^2^ = .067) predicted that the participants’ knowledge on ADHD (*β* = −.220; *p* < .001) and the current number of students taught who exhibit ADHD-type behaviors (*β* = .149; *p* = .004) were found to be significant predictors. The participants whose scores were higher on the knowledge test were less likely to hold *negative beliefs about ADHD*. Interestingly, the participants who had a greater number of students with ADHD behaviors were more likely to hold *negative beliefs about ADHD*.

#### Multiple Regression of Factor 5: Positive Consequences of ADHD-Type Behaviors

The regression model on the factor, *positive consequences of ADHD-type behaviors* (*R*^2^ = .101) showed that the age of participants (*β* = −.239; *p* = .009), whether or not the participants were special education/learning and support teachers (*β* = .122; *p* = .017), whether or not they had a higher degree (*β* = .094; *p* = .047) and their ADHD-specific knowledge score (*β* = .103; *p* = .035) were significant predictors.

## Discussion

### ADHD-Specific Attitudes Held by NSW Government School Teachers

The Scale of ADHD-Specific Attitudes (SASA) is aimed at determining the attitudes of school teachers toward ADHD, students who display ADHD-type behaviors, and the teaching thereof. The present study had a unique perspective when investigating teacher ADHD-specific attitudes, as the research instrument was comprised of a sufficiently large number of items to adequately assess ADHD-specific attitudes, did not use items that were factual questions, and not only investigated teacher attitudes toward the teaching of student with ADHD, but also investigated a broader view of teacher ADHD-specific attitudes.

In New South Wales, the education system is governed by the overarching Alice Springs (Mparntwe) Education Declaration, which ensures all Australian governments are committed to “promoting excellence and equity in Australian education” (Education Council, 2019), thus striving for inclusive education for all students. Under federal legislation ADHD and its associated behaviors (diagnosed or imputed) are classified as a disability (Commonwealth Government of Australia, 2006) and, as such, must be catered for in all educational activities and environments. The current study found that although over 90% of participants agreed (to varying degrees) that ADHD is a valid diagnosis, just under 50% indicated that they believed that ADHD was over diagnosed, and just over a third agreed that students who display ADHD-type behaviors need more structure and discipline, not assistance with their academic work. Given the legislative requirement to adjust and accommodate for students with ADHD (diagnosed or imputed) (Commonwealth Government of Australia, 2006) these are concerning statistics, as attitude has a direct impact on teacher behavior in the classroom and the kinds of supports and interventions provided to students who display ADHD-type behaviors.

As the current study presented a somewhat unique view of teacher ADHD-specific attitudes (not merely interpreting misinformation/misperceptions as attitudes or using a traditional model of attitude), it was difficult to draw many meaningful comparisons between this work and the existing literature. A variety of previous studies focused on specific facets of ADHD-specific attitudes, which provided a basis for some comparative discussion. The current study found that more than two-thirds of teachers agreed to varying degrees that students who display ADHD-type behaviors caused them to experience stress. This supported research by Greene et al. (2002), who found that the global stress and the stress scores for all subscales were higher when teaching students with ADHD, and that students who exhibited more extrinsic behavior caused more stress to teachers than those who exhibited intrinsic behaviors. Interestingly, the percentage of participants who agreed (to some extent) that students who display ADHD-type behaviors caused them to experience stress in the present study was approximately 2.5% less than the results in Mulholland et al. (2015). This could be due to several factors including, but not limited to, increased access to ADHD-specific professional learning and an increased presence in mainstream schools of learning and support specialist staff. In addition to causing teachers to feel stress, just under two-thirds of the participants in the present study indicated that students who display ADHD-type behaviors interfered with their ability to effectively teach their classes (increased from that found by Mulholland et al., 2015) and a quarter of the participants indicated that they disliked teaching classes that contain students who exhibit ADHD-type behaviors (decreased from Mulholland et al.). These results highlight that teaching students who display ADHD-type behavior significantly impacts teacher wellbeing, an area of increasing focus (Hascher & Waber, 2021) and that is vital to address given the significant teacher shortage gripping NSW (Farid, 2022).

Several studies have investigated teacher ADHD-specific attitudes using a traditional theoretical framework of attitude. These studies designed their research instruments to investigate teacher attitudes toward teaching students with ADHD and offer insight into a narrow view of attitudes. Although the current study was designed to have a scope beyond this view, these studies are useful in the broader discussion of ADHD-specific attitudes held by education professionals. Kos (2008) found teachers strongly disagreed that managing the behaviors of students with ADHD was easy, however, the current study contradicted this, with almost 60% of participants agreeing that accommodations for students with ADHD-type behaviors are easy to implement in a general education classroom, and only 3% strongly disagreed. These results are pleasing, given the focus on inclusive education and the implementation of interventions to support positive student behavior in NSW (NSW Department of Education, 2022). Kos and the current study found commensurate results with respect to teacher beliefs about behavior and ADHD with teachers typically indicating they feel that the behavior of student with ADHD is not a choice, and not because the children are naughty. Martinussen et al. (2011) found that participants reported receiving inadequate ADHD-specific training. Furthermore, findings by Ward et al. (2021) suggested that teaching staff desired professional learning that included information to increase their ADHD-specific knowledge and practical strategies to support students with ADHD-type behaviors. These results were echoed by the current study, which found that over 90% of participants wanted to know more about ADHD, its associated behavior, and classroom interventions that support them in educating students who displayed ADHD-type behaviors. Overall, the present study found that the participants were relatively positive about ADHD, students who exhibit ADHD-type behavior, and the teaching thereof, believing that ADHD is a valid diagnosis (92% of respondents) and that students who exhibit ADHD-type behaviors are rewarding to work with (71% of respondents agreed to some extent (an aggregate of the varying degrees of agree). However, participants were somewhat negative about students with ADHD in their classroom, citing that students who display ADHD-type behaviors are challenging to teach (86% of participants agreed to some degree), a result commensurate with results found by Mulholland et al. (2015). Interestingly, while 70% of respondents agreed (to some degree) that ADHD-type behaviors are irritating, this is a decrease of 8% compared to results found by Mulholland et al. (2015).

### Socio-Demographic Predictors of ADHD-Specific Attitudes

In addition to determining the ADHD-specific attitudes held by NSW Government school teachers, the present study also endeavored to investigate the influence, if any, of the socio-demographic features of teachers on their level of ADHD-specific attitudes, and if these features had any predictive value. This approach of investigating teacher ADHD-specific attitudes is somewhat novel.

The previous research that investigated demographic features of teachers as predictors of their ADHD-specific attitudes, predominantly used either correlation or means testing as the only analytical technique to determine the predictive value of the socio-demographic data. None of these studies found any socio-demographic predictors of attitude.

To investigate predictors of ADHD-specific attitudes, highly correlated items in the SASA were grouped together into factors (themes) using exploratory factor analysis (EFA). A variety of different analytical techniques were then employed to determine if the socio-demographic data had a significant influence on how a participant responded to items in the factors. EFA found, through the extraction of factors, the underlying themes from the SASA were *negative consequences of ADHD-type behaviors, desire for professional learning, feeling of knowledgeability, negative beliefs about ADHD*, and *positive consequences of ADHD-type behaviors*. It was difficult to make significant comparisons between the current research and previous research in this area, as much of the previous research created global attitude scores, had different items to determine teacher ADHD-specific attitudes, or grouped the items in different themes. The factor structure that emerged through EFA was similar to what was expected. It was expected that a sixth factor, *positive beliefs about ADHD*, would also be extracted, which would include items such as “Students who exhibit behaviors associated with ADHD perform well in some subjects and not others.” However, some of the items expected to form this factor were omitted from the factor structure completely or formed part of another factor *positive consequences of ADHD-type behaviors.* A variety of socio-demographic data was significantly correlated with each factor, and the type of school (school for specific purposes or other school type) in which the participants worked was significantly correlated with all five factors.

Of the studies that discussed socio-demographic features of teachers as an influential factor of ADHD-specific attitudes, some simply made a comparison of scores between participants in different countries (e.g., Jerome et al., 1994; Norvilitis & Fang, 2005). Some studies (e.g., Bekle, 2004; Liang & Gao, 2016) discussed the difference in attitude between pre-service and in-service teachers, reporting no significant difference between the two groups. This result can be extrapolated to the experience of the participants. The current study examined the influence of experience on the ADHD-specific attitudes of participants, finding that experience was not significantly correlated with four of the five “themes” that emerged from the SASA. There was a moderately weak, negative correlation between the factor *positive consequences of ADHD-type behaviors*, suggesting that as teachers gain more experience they are less likely to agree with statements relating to this factor (or theme). Interestingly, older teachers are more likely to agree with *negative consequences of ADHD-type behaviors* and disagree with *positive consequence of ADHD-type behaviors*. The researchers posited that this could be due to the notion that as a teacher gets older, their tolerance for behavior outside the norm is reduced, although the literature suggests that the correlation between depersonalization and misbehavior is lower for older teachers (Aloe et al., 2014).

Previous research into the differences in ADHD-specific attitudes between gender (e.g., Ghanizadeh et al., 2006) found that there were no significant differences. In contrast, the present study found that there was a significant difference in the attitudes related to n*egative beliefs about ADHD*, with female participants less likely to agree with items in this factor than male participants. This indicated that female teachers are less likely to find ADHD-type behaviors irritating and are less likely to experience stress due to these behaviors. These results contradicted Alter et al. (2013), who found that verbal interruptions and off-task behavior (behaviors often associated with ADHD) were rated as more problematic by female teachers than male teachers. Additionally, the present study found that female teachers were more likely to be able to effectively teach their class, despite ADHD-type behaviors being exhibited in their class.

In NSW, students who exhibit ADHD-type behaviors are generally educated in mainstream settings, however, due to its comorbidity many students in support classes (special education classes within a mainstream school) and schools for specific purposes (SSPs; special education schools) have an ADHD diagnosis. There was a significant difference between the attitudes of special educators (those who work in support classes or SSPs) and learning support teachers (qualified teachers who specialize in supporting students with disability and challenging behaviors in mainstream educational settings); and teachers of other subjects for four of the five factors. Overall, special educator/learning support teachers are more favorable toward and feel more knowledgeable about ADHD than teachers of other subject areas. Greenway and Rees Edwards (2020) found related results with teaching assistants holding more favorable attitudes toward students with ADHD-type behaviors than teachers. While the results are not strictly comparable as teaching assistants are paraprofessionals, whereas special educators and learning and support teachers (in the present study) were qualified teachers, together they indicated that education staff who specialize in working with students with disability including those with ADHD have a more favorable attitude toward them.

Teachers who had taught students with ADHD-type behaviors felt less knowledgeable about ADHD and the effective management of ADHD-type behaviors than those who had not. This may indicate that, by teaching a student with ADHD-type behavior, teachers notice gaps in their knowledge when their knowledge of behavior management and ADHD is tested. The present research also found that teachers who had taught a student with an ADHD diagnosis were less likely to agree with statements about negative consequences of ADHD than those participants who had taught students who displayed ADHD-type behaviors yet did not have a diagnosis. This contradicts previous research (Ohan et al., 2011) that examined the impact of an ADHD label on teacher perceptions. The present research suggested that the diagnosis, and therefore the “label,” had the potential to impact how an educator views ADHD-type behaviors in the classroom and hence their behavior in the classroom including the implementation of positive strategies and interventions.

In addition to investigating the influence of the socio-demographic data on ADHD-specific attitudes, this study also examined the influence of participant ADHD-specific knowledge on their attitudes. Previous research (Bekle, 2004; Ghanizadeh et al., 2006) found that there was a significant correlation between participant ADHD-specific knowledge and their ADHD-specific attitudes, however, more recent research (Blotnicky-Gallant et al., 2015; Youssef et al., 2015) contradicted this. The present study found that ADHD-specific knowledge was significantly positively correlated with *feeling of knowledgeability* and *positive consequences of ADHD-type behaviors* and significantly negatively correlated with *negative beliefs about ADHD*. This suggests that the more knowledge a participant had, the more likely they were to feel knowledgeable, to agree with positive statements about the consequences of ADHD-type behaviors, and to disagree with negative beliefs about ADHD.

Positively, NSW government schoolteachers now have access to an OnLine Training Course titled *Understanding Attention Deficit/Hyperactivity Disorder* aimed at increasing teacher ADHD-specific knowledge and strategies to support students who display ADHD-type behaviors. However, this course has only a small number of trainers across the state and a maximum cohort size of 12 people (OnLine Training Ltd, 2021). The results of the present study suggest that participation in this course may improve teacher ADHD-specific attitudes resulting (based on the theoretical connection between attitudes and behavior) in a positive impact on the implementation of supports for these students.

The predictive power of these models derived from data in this study is somewhat limited, as this model accounted for only a small amount of the variation (between 0.7% and 14.7% of the variance) of ADHD-specific attitudes among all the study participants. This suggests that there are a variety of other factors that have an impact on teacher ADHD-specific attitudes outside the scope of this thesis. These factors could include personal experience of people with ADHD, ADHD-specific personal and professional learning, class size, and participant personality.

## Conclusion

This study illustrated that teachers had generally positive attitudes toward students who display ADHD-type behaviors, however, they found the externalized behaviors of ADHD irritating in the classroom, and found teaching students with ADHD-type behaviors difficult, as they often lack the knowledge about classroom strategies to support them. Further, it was shown that teachers overwhelmingly want more information about ADHD and how to manage ADHD-type behaviors in the classroom, pointing to a need for the development of professional learning modules and more support from the Department of Education for teachers in the area of ADHD. This paper provided evidence to suggest that the Theory of Planned Behavior (TPB) does not provide a strong basis for a comprehensive and in-depth analysis of attitudes toward disability due to the multi-faceted nature of attitude itself. As such, a research instrument based on a new approach to a theoretical framework of attitude was used. The exploratory factor analysis of teachers’ attitudes toward ADHD, students who exhibit ADHD-type behaviors and the teaching thereof, revealed a five-factor solution: *negative consequences of ADHD-type behaviors, desire for professional learning, feeling of knowledgeability, negative beliefs about ADHD, positive consequences of ADHD-type behaviors*. Finally, the multiple regression analyses showed that the socio-demographic data provided by the participants (including ADHD-specific knowledge level) are not good predictors of teacher ADHD-specific attitudes, suggesting that further professional learning is needed in the area of ADHD across the teaching profession.

## Limitations

There are several limitations to this study that could have an impact on further research in this area. One methodological limitation of this study is that teachers’ participation was voluntary. As teachers self-selected to participate in this study, there may have been some inherent differences between the ADHD-specific knowledge and attitudes of those who chose to participate and those who did not.

A second methodological limitation of this study is the scope of demographic predictors surveyed. The scope of the socio-demographic data was limited to ensure that the research instrument was not prohibitively time consuming and thus maximized participation and completion rates. The final analysis showed that, although some of the socio-demographic data collected were significant predictors of teacher ADHD-specific attitudes, they accounted for very little of the variance in ADHD-specific knowledge and attitudes. This indicated that other variables may be associated with teacher ADHD-specific knowledge and attitudes. The authors hypothesize that variables such as personal family experience with ADHD, severity of student ADHD-type behavior, country of initial teacher education, and teacher personality and temperament could have a statistically significant impact on knowledge and attitudes of ADHD, thus further research in this area is needed.

The final methodological limitation of this study was the potential for experiment-wise errors to occur, given the number of analytical techniques applied to the data. However, in the current dataset, there were only a few socio-demographic variables that were significant at the conventional alpha level. Thus, committing Type I errors was not particularly concerning, as most statistical testing using the conventional alpha level, yielded non-significant results and therefore, statistical adjustments of significance levels would not lead to different results and would lead to Type II errors.

## Implications for Practice and Directions for Future Research

A variety of legislation and NSW Department of Education policies outline that every student deserves a meaningful and appropriate education. It is widely known that knowledge and attitudes of teachers directly impact their behavior in the classroom (Avramidis & Norwich, 2002; Cronin-Jones, 1991; Kos et al., 2006; Leatherman & Niemeyer, 2005), therefore professional learning is vital to providing equitable and meaningful education to students who display ADHD-type behaviors. While the present study found that the significant predictors of teacher ADHD-specific knowledge only accounted for a small amount of variation, it did however, find that teachers overwhelmingly want to know more about ADHD and strategies to manage ADHD-type behavior is the classroom. This has some significant implications for the professional learning provided at both the school and system level. It is recommended that individual schools seek appropriate professional learning about ADHD from an educational professional with expert knowledge in the area. It is also recommended that the NSW Department of Education investigate large scale methods of delivery for ADHD-specific professional learning to support as many staff as possible.

The present research focused on teachers from NSW government schools. Further research into teacher ADHD-specific attitudes could include both Catholic and Independent schools and/or could include research of teachers from countries other than Australia to determine if ethnicity and/or school educational ethos is a significant predictive factor. The current study found only weak to moderate correlations between the various teacher demographics and ADHD-specific attitudes, and the regression models accounted for a small percent of the variance. As such, further research should include independent variables such as time constraints, class size, available resources, severity of student behavior, teachers’ familial experience of children with ADHD, and the extent to which teacher have engaged in ADHD-specific professional learning.

In addition to investigating additional demographic characteristics, further work could include the examination of student and parent perceptions of their teacher’s ADHD-specific knowledge and attitudes. As literature suggests knowledge and attitudes have a direct impact on teacher behavior in the classroom (Kos et al., 2006), this could provide valuable insight into the relationship between students who display-ADHD type behaviors and their parents, and their teachers.

## References

[bibr1-10870547231153938] Ajzen I. (1985). From intention to actions: A theory of planned behavior. In Kulh J. Beckmann J. (Eds.), Action-control: From cognition to behavior (pp. 11–39). Springer.

[bibr2-10870547231153938] Ajzen I. (2001). Nature and operation of attitudes. Annual Review of Psychology, 52, 27–58. 10.1146/annurev.psych.52.1.27 11148298

[bibr3-10870547231153938] Alabd A. M. A. Mesbah S. K. Alboliteeh M. (2018). Effect of educational program on elementary school teachers’ knowledge, attitude, and classroom management techniques regards attention deficit hyperactivity disorder. International Journal of Nursing Studies, 3(3), 159–171. 10.20849/ijsn.v3i3.528

[bibr4-10870547231153938] Alanazi F. Al Turki Y. (2021). Knowledge and attitude of attention-deficit and hyperactivity disorder (ADHD) among male primary school teachers, in Riyadh City, Saudi Arabia. Journal of Family Medicine and Primary Care, 10, 1218–1226. 10.4103/jfmpc.jfmpc_2194_20 PMC8140244 34041155

[bibr5-10870547231153938] Aloe A. M. Amo L. C. Shanahan M. E. (2014). Classroom management self-efficacy and burnout: A multivariate meta-analysis. Educational Psychology Review, 26, 101–126.

[bibr6-10870547231153938] Alter P. Walker J. Landers E. (2013). Teachers’ perceptions of students’ challenging behavior and the impact of teacher demographics. Education and Treatment of Children, 36(4), 51–69. 10.1353/etc.2013.0040

[bibr7-10870547231153938] American Psychiatric Association. (2022). Diagnostic and statistical manual of mental disorders (5th text revision ed.).

[bibr8-10870547231153938] Amha H. Azale T. (2022). Attitudes of primary school teachers and its associated factors toward students with attention deficit hyperactivity disorder in Debre Markos and Dejen towns, Northwest Ethiopia. Frontiers in Pediatrics, 10, 805440. 10.3389/fped.2022.805440 35601433 PMC9120593

[bibr9-10870547231153938] Anderson D. L. Watt S. E. Noble W. Shanley D. C. (2012). Knowledge of attention deficit hyperactivity disorder (ADHD) and attitudes toward teaching children with ADHD: The role of teaching experience. Psychology in the Schools, 49, 511–525. 10.1002/pits.21617

[bibr10-10870547231153938] Australian Government National Health and Medical Research Council. (2018, July). National statement on ethical conduct in human research (2007) - Updated March 2014. https://www.nhmrc.gov.au/about-us/publications/national-statement-ethical-conduct-human-research-2007-updated-2018#block-views-block-file-attachments-content-block-1

[bibr11-10870547231153938] Avramidis E. Norwich B. (2002). Teachers’ attitudes towards integration/inclusion: A review of the literature. European Journal of Special Needs Education, 17, 129–147. 10.1080/08856250210129056

[bibr12-10870547231153938] Barkley R. A. (2015). Attention-deficit hyperactivity disorder: A handbook for diagnosis and treatment (4th ed.). The Guilford Press.

[bibr13-10870547231153938] Bekle B. (2004). Knowledge and attitudes about attention-deficit hyperactivity disorder (ADHD): A comparison between practicing teachers and undergraduate education students. Journal of Attention Disorders, 7, 151–161. 10.1177/108705470400700303 15260172

[bibr14-10870547231153938] Bell L. Long S. Garvan C. Bussing R. (2011). The impact of teacher credentials on ADHD stigma perceptions. Psychology in the Schools, 48, 184–197. 10.1002/pits.20536

[bibr15-10870547231153938] Blotnicky-Gallant P. Martin C. McGonnell M. Corkum P. (2015). Nova Scotia teachers’ ADHD knowledge, beliefs, and classroom management practices. Canadian Journal of School Psychology, 30, 3–21. 10.1177/0829573514542225

[bibr16-10870547231153938] Breen G. (2022, October 5). ADHD guidelines have finally been released in Australia. Here’s why that matters. https://www.abc.net.au/news/2022-10-05/adhd-guidelines-australia-released/101500966

[bibr17-10870547231153938] Carroll J. M. Maughan B. Goodman R. Meltzer H. (2005). Literacy difficulties and psychiatric disorders: Evidence for comorbidity. Journal of Child Psychology and Psychiatry, 46, 524–532. 10.1111/j.1469-7610.2004.00366.x 15845132

[bibr18-10870547231153938] Commonwealth Government of Australia. (2006). Disability standards of education 2005.

[bibr19-10870547231153938] Cronin-Jones L. L. (1991). Science teacher beliefs and their influence on curriculum implementation: Two case studies. Journal of Research in Science Teaching, 28(3), 235–250.

[bibr20-10870547231153938] Cueli M. Areces D. Rodríguez C. Cabaleiro P. González-Castro P. (2022). Differences between Spanish students’ and teaching professionals’ knowledge of and attitudes toward ADHD—Does knowledge influence attitude? Psychology in the Schools, 59(2), 242–259. 10.1002/pits.22605

[bibr21-10870547231153938] Dessie M. Techane M. A. Tesfaye B. Gebeyehu D. A. (2021). Elementary school teachers knowledge and attitude towards attention deficit-hyperactivity disorder in Gondar, Ethiopia: A multi-institutional study. Child and Adolescent Psychiatry and Mental Health, 15(1), 16. 10.1186/s13034-021-00371-9 33827642 PMC8028709

[bibr22-10870547231153938] Dort M. Strelow A. E. Schwinger M. Christiansen H. (2020). Working with children with ADHD—A latent profile analysis of teachers’ and psychotherapists’ attitudes. Sustainability, 12, 9691. 10.3390/su12229691

[bibr23-10870547231153938] Education Council. (2019). Alice springs (Mparntwe) education declaration. https://docs.education.gov.au/documents/alice-springs-mparntwe-education-declaration

[bibr24-10870547231153938] Farid F. (2022, August 4). NSW teacher shortage is a crisis: Union. The Maitland Mercury. https://www.maitlandmercury.com.au/story/7846844/nsw-teacher-shortage-is-a-crisis-union/

[bibr25-10870547231153938] Field A. (2009). Discovering statistics using SPSS (3rd ed.). London: Sage.

[bibr26-10870547231153938] Flavian H. Uziely E. (2022). Determinants of teachers’ attitudes toward inclusion of pupils with attention deficit hyperactivity disorder: The role of teacher education. Frontiers in Education, 7, 1–9. 10.3389/feduc.2022.941699

[bibr27-10870547231153938] Ghanizadeh A. Bahredar M. J. Moeini S. R. (2006). Knowledge and attitudes towards attention deficit hyperactivity disorder among elementary school teachers. Patient Education and Counseling, 63, 84–88. 10.1016/j.pec.2005.09.002 16504452

[bibr28-10870547231153938] Greene R. W. Beszterczey S. K. Katzenstein T. Park K. Goring J. (2002). Are students with ADHD more stressful to teach? Patterns of teacher stress in an elementary school sample. Journal of Emotional and Behavioral Disorders, 10, 79–89. 10.1177/10634266020100020201

[bibr29-10870547231153938] Greenway C. W. Rees Edwards A. (2020). Knowledge and attitudes towards attention-deficit hyperactivity disorder (ADHD): A comparison of teachers and teaching assistants. Australian Journal of Learning Difficulties, 25(1), 31–49. 10.1080/19404158.2019.1709875

[bibr30-10870547231153938] Hair J. F. Black W. C. Babin B. J. Anderson R. E. (2010). Multivariate data analysis: A global perspective. Upper Saddle River, NJ: Pearson.

[bibr31-10870547231153938] Hascher T. Waber J. (2021). Teacher well-being: A systematic review of the research literature from the year 2000–2019. Review of Educational Research, 34, 100411. 10.1016/j.edurev.2021.100411

[bibr32-10870547231153938] Jerome L. Gordon M. Hustler P. (1994). A comparison of American and Canadian teachers’ knowledge and attitudes towards attention deficit hyperactivity disorder (ADHD). The Canadian Journal of Psychiatry, 39, 563–567. 7874659 10.1177/070674379403900909

[bibr33-10870547231153938] Jerome L. Washington P. Laine C. J. Segal A. (1999). Graduating teachers’ knowledge and attitudes about attention-deficit hyperactivity disorder: A comparison with practicing teachers. The Canadian Journal of Psychiatry, 44, 192. 10097848

[bibr34-10870547231153938] Kos J. M. (2008). What do teachers know, think and intend to do about ADHD? Australian Council for Educational Research. http://research.acer.edu.au/tll_misc/9

[bibr35-10870547231153938] Kos J. M. Richdale A. L. Hay D. A. (2006). Children with attention deficit hyperactivity disorder and their teachers: A review of the literature. International Journal of Disability Development and Education, 53, 147–160. 10.1080/10349120600716125

[bibr36-10870547231153938] Leatherman J. Niemeyer J. (2005). Teachers’ attitudes toward inclusion: Factors influencing classroom practice. Journal of Early Childhood Teacher Education, 26, 23–36. 10.1080/10901020590918979

[bibr37-10870547231153938] Liang L. Gao X. (2016). Pre-service and in-service secondary school teachers’ knowledge about attention-deficit hyperactivity disorder (ADHD) and attitudes toward students with ADHD. International Journal of Disability Development and Education, 63, 369–383. 10.1080/1034912X.2015.1123231

[bibr38-10870547231153938] Mahar P. Chalmers L. (2007). Teachers’ perceptions of students diagnosed with ADHD. National Forum of Applied Educational Research Journal, 20(3), 1-8.

[bibr39-10870547231153938] Martinussen R. Tannock R. Chaban P. (2011). Teachers’ reported use of instructional and behavior management practices for students with behavior problems: Relationship to role and level of training in ADHD. Child & Youth Care Forum, 40, 193–210. 10.1007/s10566-010-9130-6

[bibr40-10870547231153938] Mulholland S. (2016). ADHD-specific knowledge and attitudes of teachers (ASKAT): Development and validation of a new research instrument. International Journal of Educational Research, 77, 109-116. 10.1016/j.ijer.2016.03.010

[bibr41-10870547231153938] Mulholland S. M. Cumming T. M. Jung J. Y. (2015). Teacher attitudes towards students who exhibit ADHD type behaviours. Australasian Journal of Special Education, 39, 15-36. 10.1017/jse.2014.1

[bibr42-10870547231153938] Mulholland S. Cumming T. Lee J. (2022). Assessment of Teachers’ knowledge of attention deficit hyperactivity disorder (ADHD) [Manuscript submitted for publication]. School of Education, University of NSW.

[bibr43-10870547231153938] Norvilitis J. M. Fang P. (2005). Perceptions of ADHD in China and the United States: A preliminary study. Journal of Attention Disorders, 9, 413–424. 10.1177/1087054705281123 16371664

[bibr44-10870547231153938] NSW Department of Education. (2022). Personalised learning and support for students roles and responsibilities. https://education.nsw.gov.au/teaching-and-learning/disability-learning-and-support/personalised-support-for-learning/roles-and-responsibilities#Learning2

[bibr45-10870547231153938] Ohan J. L. Visser T. A. Strain M. C. Allen L. (2011). Teachers’ and education students’ perceptions of and reactions to children with and without the diagnostic label “ADHD”. Journal of School Psychology, 49, 81–105. 10.1016/j.jsp.2010.10.001 21215837

[bibr46-10870547231153938] Olivier M. A. J. Steenkamp D. S. (2004). Attention-deficit/hyperactivity disorder: Underlying deficits in achievement motivation. International Journal for the Advancement of Counseling, 26, 47–63. 10.1023/b:adco.0000021549.40409.c4

[bibr47-10870547231153938] OnLineTraining Ltd. (2021, August 13). New understanding attention deficit/hyperactivity disorder course. OLT Australia. https://aus.oltinternational.net/news-item/New-Understanding-Attention-637644687519317990

[bibr48-10870547231153938] Rodrigo M. D. Perera D. Eranga V. P. Williams S. S. Kuruppuarachchi K. A. (2011). The knowledge and attitude of primary school teachers in Sri Lanka towards childhood attention deficit hyperactivity disorder. Ceylon Medical Journal, 56, 51–54. 21789864 10.4038/cmj.v56i2.3108

[bibr49-10870547231153938] Sciutto M. J. Terjesen M. D. Frank A. S. B. (2000). Teachers’ knowledge and misperceptions of attention-deficit/hyperactivity disorder. Psychology in the Schools, 37(2), 115–122. 10.1002/(sici)1520-6807(200003)37:2<115::aid-pits3>3.0.co;2-5

[bibr50-10870547231153938] Soriano Ferrer M. Echegaray-Bengoa J . (2021). Knowledge and beliefs about ADHD of Peruvian teachers: The role of teaching experience with ADHD. MLS Educational Research, 5(1), 25–46. 10.29314/mlser.v5i1.277

[bibr51-10870547231153938] van Aalderen-Smeets S. I. Walma van der Molen J. H. Asma L. J. F . (2012). Primary teachers’ attitudes toward science: A new theoretical framework. Bioscience Education, 96, 158–182. 10.1002/sce.20467

[bibr52-10870547231153938] Ward R. J. Kovshoff H. Kreppner J. (2021). School staff perspectives on ADHD and training: Understanding the needs and views of UK primary staff. Emotional and Behavioural Difficulties, 26(3), 306–321. 10.1080/13632752.2021.1965342

[bibr53-10870547231153938] Youssef M. K. Hutchinson G. Youssef F. F. (2015). Knowledge of and attitudes toward ADHD among teachers: Insights from a Caribbean nation. Sage Open, 5, 1–8. 10.1177/2158244014566761

